# Supplementary material for: The origin of exceptionally large ductility in molybdenum alloys dispersed with irregular-shaped La2O3 nano-particles
Source: Nat Commun. 2024 May 15;15:4105. doi: 10.1038/s41467-024-48439-2 (PMC11096377; doi:10.1038/s41467-024-48439-2)
Supplement: Supplementary file 1 — Supplementary Information [file 41467_2024_48439_MOESM1_ESM.pdf]

Supplementary information

The origin of exceptionally large ductility in Molybdenum Alloys dispersed with Irregular-Shaped La_2O_3 Nano-Particles

Yujie Chen[1,2,#], Yan Fang[1,3,#], Pengming Cheng[4,#], Xiaoxing Ke[5], Manchen Zhang[5], Jiawei Zou[1], Jun Ding[6,*], Bozhao Zhang[6], Lin Gu[2,*], Qinghua Zhang[7], Gang Liu[4,*], Qian Yu[1,*]

[1]Center of Electron Microscopy and State Key Laboratory of Silicon and Advanced Semiconductor Materials, Department of Materials Science and Engineering, Zhejiang University, Hangzhou 310027, China

[2]Beijing National Center for Electron Microscopy and Laboratory of Advanced Materials, School of Materials Science and Engineering, Tsinghua University, Beijing 100084, China

[3]Department of Mechanical Engineering, The University of Hong Kong, Hong Kong 999077, China

[4]State Key Laboratory for Mechanical Behavior of Materials, School of Materials Science and Engineering, Xi'an Jiaotong University, Xi'an 710049, China

[5]Beijing Key Laboratory of Microstructure and Properties of Solids, College of Materials Science and Engineering, Beijing University of Technology, Beijing 100124, China

[6]Center for Alloy Innovation and Design, State Key Laboratory for Mechanical Behavior of Materials, Xi'an Jiaotong University, Xi'an 710049, China

[7]Beijing National Laboratory for Condensed Matter Physics, Collaborative Innovation Center of Quantum Matter, Institute of Physics, Chinese Academy of Sciences, Beijing 100190, China

#These authors contributed equally: Yujie Chen, Yan Fang, Pengming Cheng

*Correspondence should be addressed to: yu_qian@zju.edu.cn; lingu@mail.tsinghua.edu.cn; dingsn@xjtu.edu.cn; lgsammer@mail.xjtu.edu.cn.

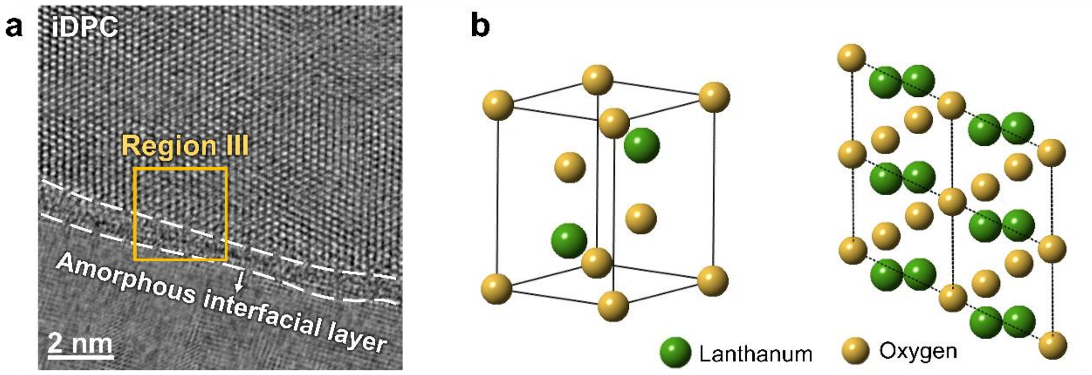

Fig. S1 The atomic-scale structure of La_2O_3 nanoparticle at the interface. a, The iDPC-STEM image showing the atomic-scale structure of the La_2O_3 nanoparticle. The high-resolution iDPC-STEM image in Fig. 1e is acquired at the location of Region III, which is marked by the orange square. **b,** The perfect crystal structure model of the La_2O_3 nanoparticle. The perfect crystal structure model of La_2O_3 has the space group of $P\bar{3}m1$.

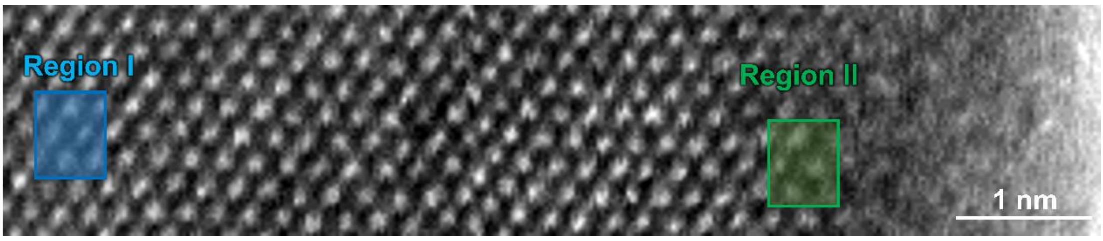

Fig. S2 HAADF-STEM image and the region where the EELS spectrum taken from. Region I (blue rectangle) is in the internal La_2O_3 particle. Region II (green rectangle) is in proximity to the amorphous layer.

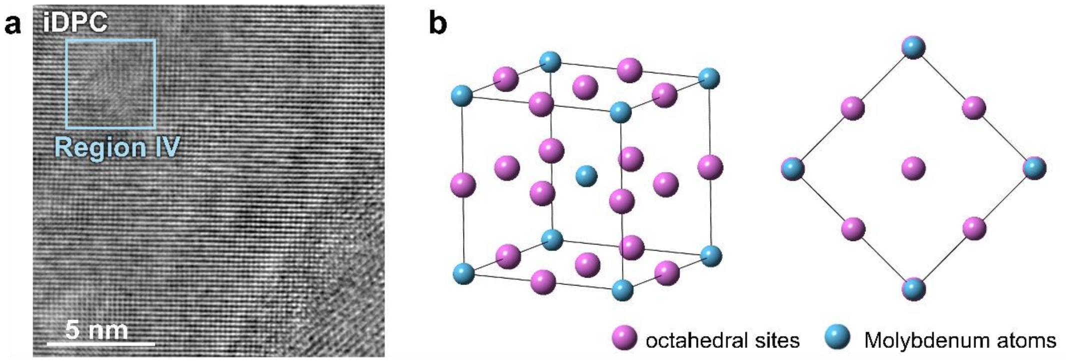

Fig. S3 The atomic-scale structure of Mo matrix near La_2O_3 nanoparticle. a, The iDPC-STEM image showing the aggregation of oxygen atoms near the particle-matrix interface in the Mo matrix. Region IV (marked by light blue square) is the typical cluster of oxygen atoms, which is magnified as Fig. 1f. **b,** The crystal structure model of the Mo matrix, with the oxygen atoms occupy octahedral interstitial sites.

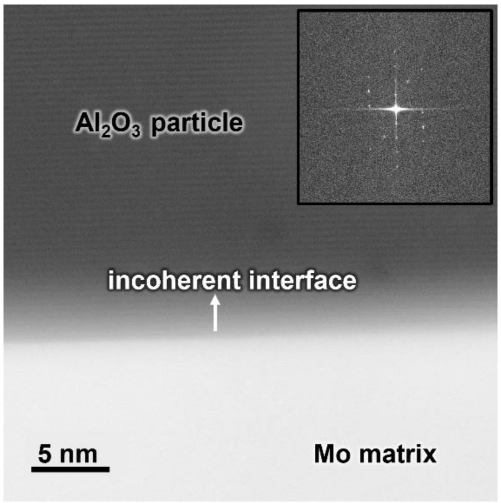

Fig. S4 HAADF-STEM image of the interface between Al_2O_3 particle and Mo matrix in Mo-Al_2O_3 alloy. The atomic-resolution image of this interface displays incoherent and the corresponding fast Fourier Transform image (inserted in the top right) also shows a typical incoherent crystalline interface characteristic.

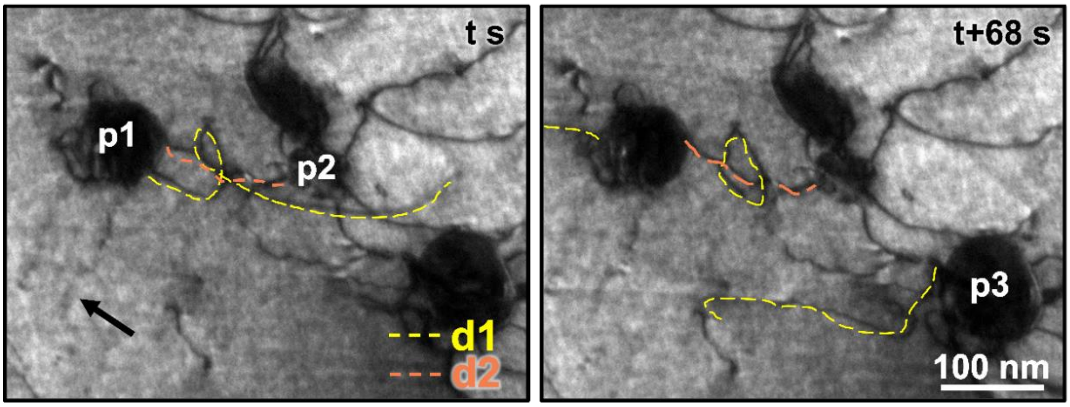

Fig. S5 Dislocation interactions at room temperature with the participation of particle. Particle 1 and 2 are marked by p1, and p2, respectively. Dislocation 1 and 2 (marked by yellow and orange dashed lines) were pinned by particle 1 and 2 (marked as p1 and p2), respectively. Their interaction produced a dislocation loop and two single armed dislocations attached to p1 and particle 3 (marked as p3), respectively.

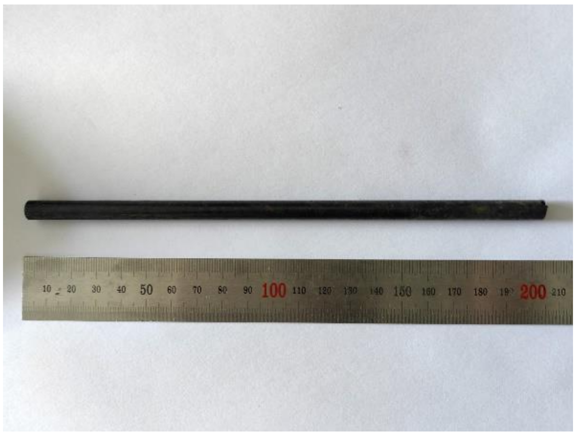

Fig. S6 A rod of Mo-La_2O_3 alloy after the process of rotary swaging. The diameter of the rod is approximately 8 mm.

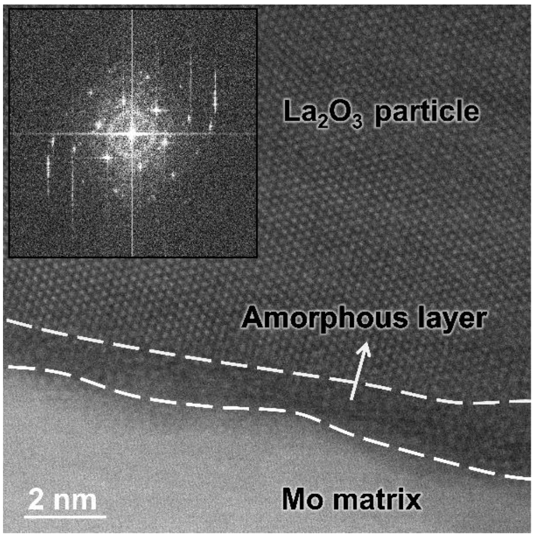

Fig. S7 HAADF-STEM and the corresponding FFT (inserted in the top left) images of the amorphous interface in Mo-La$_2$O$_3$ alloy after the treatment of rotary-swaging. An ultrathin amorphous layer at the particle-matrix interface is shown, with a measured thickness of approximately 1.5 nanometers. The fast Fourier Transform image also show a typical amorphous ring characteristic indicative of its amorphous nature.

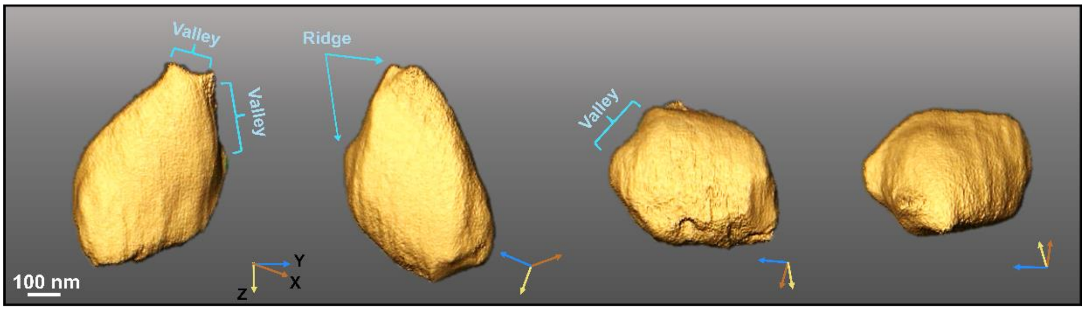

Fig. S8 The three-dimensional tomography of the La$_2$O$_3$ particles, with the long axis of approximately 500 nm. The irregular-shaped particles have considerable ridges and valleys at the interface, as marked by blue arrows.

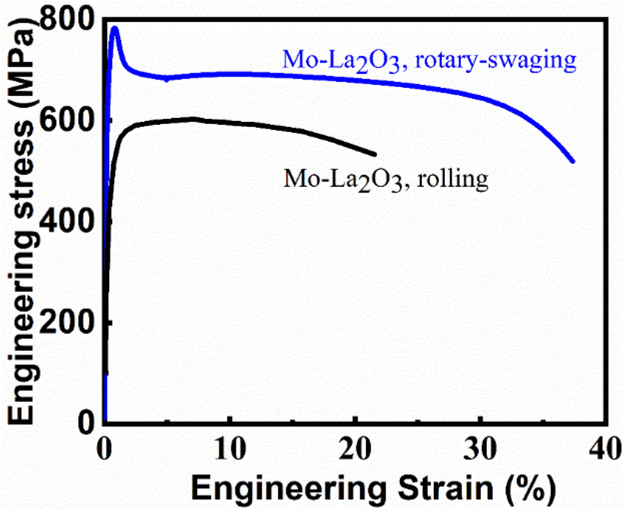

Fig. S9 Mechanical properties of Mo-La₂O₃ alloy after different treatment. Blue curve: rotary-swaging. Black curve: rolling[14].

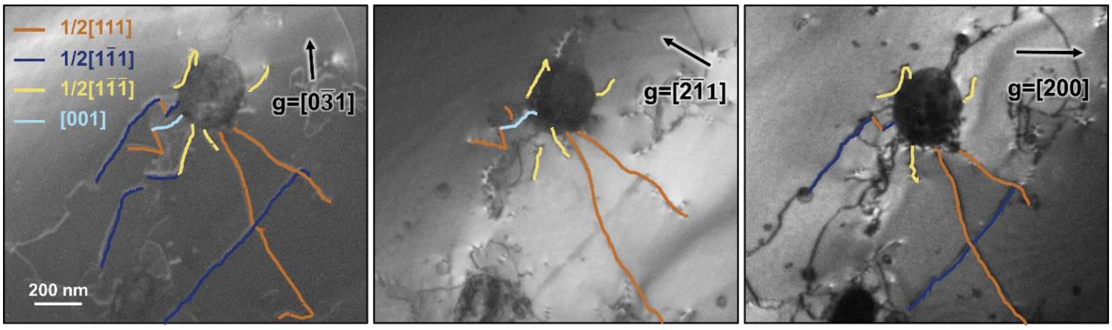

Fig. S10 Two-Beam dark-field TEM image with different diffraction vectors. Diffraction vector, **g**, annotating in each image with the direction shown by the black arrow.

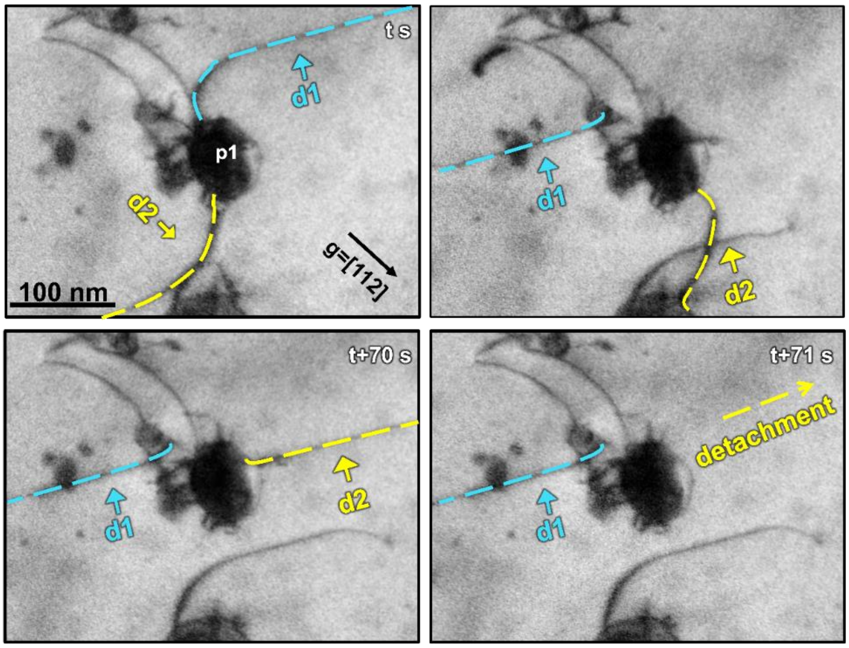

Fig. S11 In-situ TEM straining experiments on the Mo-La_2O_3 alloy processed by rotary swaging. Dislocation 1 and 2 (marked as d1 and d2 by blue and yellow dashed lines, respectively) are first pinned at the interface of particle 1 (marked as p1) with one end. d1 and d2 function as single arm sources. After several cycles, d1 and d2 detach from the surface of p1 and keep gliding.
