# Supplementary material for: The origin of exceptionally large ductility in molybdenum alloys dispersed with irregular-shaped La2O3 nano-particles
Source: Nat Commun. 2024 May 15;15:4105. doi: 10.1038/s41467-024-48439-2 (PMC11096377; doi:10.1038/s41467-024-48439-2)
Supplement: Supplementary file 3 — Description of Additional Supplementary Information [file 41467_2024_48439_MOESM3_ESM.pdf]

Description of Additional Supplementary Information

Supplementary Movie 1 In-Situ TEM tensile observation on sintered Mo-La2O3 alloy at room temperature.

Supplementary Movie 2 Molecular dynamics simulation showing edge dislocations in Mo gliding across the embedded amorphous spherical MoPd particles with periodic boundary condition.

Supplementary Movie 3 Molecular dynamics simulation showing edge dislocations in Mo gliding across the embedded amorphous spherical MoPd particles with two additional notches.

Supplementary Movie 4 The three-dimensional tomography of the La2O3 particle with particle size of approximately 100 nm.

Supplementary Movie 5 The three-dimensional tomography of the La2O3 particle with particle size of approximately 400 nm.

Supplementary Movie 6 The three-dimensional tomography of the particles and dislocations accumulated at the interface.

Supplementary Movie 7 In-situ TEM tensile observation of the formation of Frank-Read dislocation sources at room temperature.

Supplementary Movie 8 In-situ TEM tensile observation of the formation of Frank-Read dislocation sources at -50 °C.
